# Mycorrhizal associations of the exotic hickory trees, *Carya laciniosa* and *Carya cordiformis*, grown in Kórnik Arboretum in Poland

**DOI:** 10.1007/s00572-018-0846-8

**Published:** 2018-06-22

**Authors:** Maria Rudawska, Tomasz Leski, Robin Wilgan, Leszek Karliński, Marta Kujawska, Daniel Janowski

**Affiliations:** Laboratory of Symbiotic Associations, Institute of Dendrology Polish Academy of Sciences, Parkowa 5, 62-035 Kórnik, Poland

**Keywords:** Hickory, Ectomycorrhizal fungi, Arbuscular mycorrhiza, Exotic trees, Juglandaceae

## Abstract

We studied mycorrhizal associations of North American *Carya laciniosa* and *Carya cordiformis* trees*,* successfully acclimated to local habitat conditions of the historic Kórnik Arboretum in Poland, in order to better understand mycorrhizal host range extensions in new environments. The root systems of *Carya* seedlings (1–3 years old), regenerated under a canopy of mature hickory trees, were analyzed using microscopic, morphological, and molecular techniques. Our results, for the first time, indicate that *C. laciniosa* and *C. cordiformis* have both arbuscular and ectomycorrhizal associations. In the cleared and stained roots of both *Carya* species, typical structures of arbuscular mycorrhizae (vesicles, arbuscules, hyphal coils, and intercellular nonseptate hyphae) were detected. On the basis of ITS rDNA sequencing, 40 ectomycorrhizal fungal taxa were revealed, with 25 on *C. laciniosa* and 19 on *C. cordiformis*. Only four fungal species (*Cenococcum geophilum* sensu lato, *Russula recondita*, *Xerocomellus cisalpinus*, *Humaria hemisphaerica*) were shared by both *Carya* species. The high number of infrequent fungal taxa found, as well as the calculated richness estimator, indicates that the real ectomycorrhizal community of *C. laciniosa* and *C. cordiformis* is probably richer. The ability of the exotic *Carya* species to form arbuscular and ectomycorrhizal linkages with native fungi could be a factor in the successful establishment of these tree species under the conditions of Kórnik Arboretum.

**Electronic supplementary material:**

The online version of this article (10.1007/s00572-018-0846-8) contains supplementary material, which is available to authorized users.

## Introduction

Arboreta are botanical gardens dedicated to the collection and cultivation of woody tree species from all over the world that are able to thrive in a given locality. Among many different objectives that arboreta meet are scientific investigations of various kinds, including plant breeding and acclimatization research. One of the largest dendrological gardens in Europe is Kórnik Arboretum, situated in Western Poland, and located around Kórnik Castle. This garden was a prime example of Italian, afterwards French international style, progressively transformed into an English landscape park, and finally an arboretum, where species of trees and other woody plants of known origin are cultivated. During the entire nineteenth century, Kórnik Arboretum was enriched with new species and varieties by the owner of the property, Count Tytus Działyński, and his heirs, Jan Działyński and Władysław Zamoyski. Currently, Kórnik Arboretum belongs to the Institute of Dendrology, Polish Academy of Sciences, and is famous for its rich collection of trees and shrubs, originating from various parts of the northern temperate zone. Particularly numerous are species representing the woody floras of East Asia and North America. With around 3000 species and varieties of coniferous and deciduous trees and shrubs, grown on an area of 53 ha, Kórnik Arboretum has plenty of tree species that are not native to Europe. Among the alien tree species successfully established in the Kórnik Arboretum is a collection of hickories (*Carya* spp.). Hickories have been imported and planted in Europe since the seventeenth century, due to their highly ornamental qualities and valuable wood. The climatic requirements of most of the hickory species, however, prevented their effective cultivation in Europe (Krumm and Vítková 2016). Also in Kórnik Arboretum, from nine species and varieties repeatedly imported as seeds from Western European nurseries in the nineteenth century, successful acclimatization was achieved for only three species, *C. laciniosa*, *C. ovata*, and *C. cordiformis*. On the basis of long-standing observations of their developmental cycles, these three *Carya* spp. have been acknowledged as being well adapted to the local habitat conditions (Chylarecki 1961).

Hickories, as with the majority of trees, need mutualistic mycorrhizal associations to establish, grow, reproduce, and survive (Smith and Read 2008). The genus *Carya* belongs to the Juglandaceae family, which is characterized by species that associate with both ectomycorrhizal (EM) and arbuscular mycorrhizal (AM) fungi (Wang and Qiu 2006; Taber et al. 1982), except for *Juglans nigra*, which forms only arbuscular mycorrhizae (Bainard et al. 2011; Brundrett et al. 1990; Wang and Qiu 2006). Information about the EM fungal assemblages of hickory trees is very limited, and restricted to the pecan hickory (*C. illinoinensis*) (Bonito et al. 2011; Ge et al. 2017; Marozzi et al. 2017), which is economically important to the pecan nut industry (Thompson and Conner 2012), but, due to its very low frost resistance, has not been successfully acclimatized in the temperate zone of Europe. The AM fungi of *Carya* species are even less well documented, although some information about the arbuscular mycorrhizae of pecan roots in natural, semiarid environments has been provided (Taber et al. 1982). How the mycorrhizal associations of other hickories are structured has not been determined, neither from North America nor from outside their natural distribution ranges in arboreta or plantations. Introduced tree species, outside their natural range, often have to cope with a lack of native EM fungal symbionts, and could form novel associations with local mutualists that are able to replace those from their natural habitats. Recently, several authors have intensively studied this issue in forest plantations of different exotic tree species grown outside their native ranges (e.g., Bahram et al. 2013; Lothamer et al. 2014; Nuñez and Dickie 2014; O’Hanlon and Harrington 2012; O’Hanlon et al. 2013; Richardson et al. 2000; Tedersoo et al. 2007; Trocha et al. 2012; Walbert et al. 2010). In contrast to these studies on forest plantations, investigations on EM fungal assemblages of trees from arboreta remain scarce (Healy et al. 2016), especially records based on molecular analysis of their mycorrhizae.

In this study, we aimed to determine how the mycorrhizal communities of alien trees, grown under cultivation in Kórnik Arboretum, and surrounded by many other native and alien species, were structured. As a case study, we investigated the mycorrhizal associations of two exotic *Carya* species, *C. laciniosa* and *C. cordiformis*, both well adapted to conditions at Kórnik Arboretum.

Previous research has shown that trees planted outside of their native ranges host relatively species-poor EM fungal assemblages (Bahram et al. 2013; Dickie et al. 2010; Nuñez et al. 2009; Tedersoo et al. 2007; Walbert et al. 2010). Therefore, we hypothesize that hickories grown in our arboretum environment would support (1) a limited number of EM symbionts, with (2) a predominance of common and native-to-Europe generalist fungi.

During the early period of introduction of exotic trees to Kórnik Arboretum, there was a considerable, intentional intercontinental movement of soil (Białobok 1960), potentially containing mycorrhizal propagules. Based on this assumption, we predicted that (3) hickory roots harbor fungal species that are native to North America but alien to Europe.

To our knowledge, this is the first study of the EM fungal community structure of *C. laciniosa* and *C. cordiformis*. We wondered (4) if the structure of EM fungal communities in the tested hickory species would correspond to the structure of *C. illinoinensis* communities, which have been examined in *Carya*’s natural distribution area (Bonito et al. 2011).

## Material and methods

### Site description

**Fig. 1:**
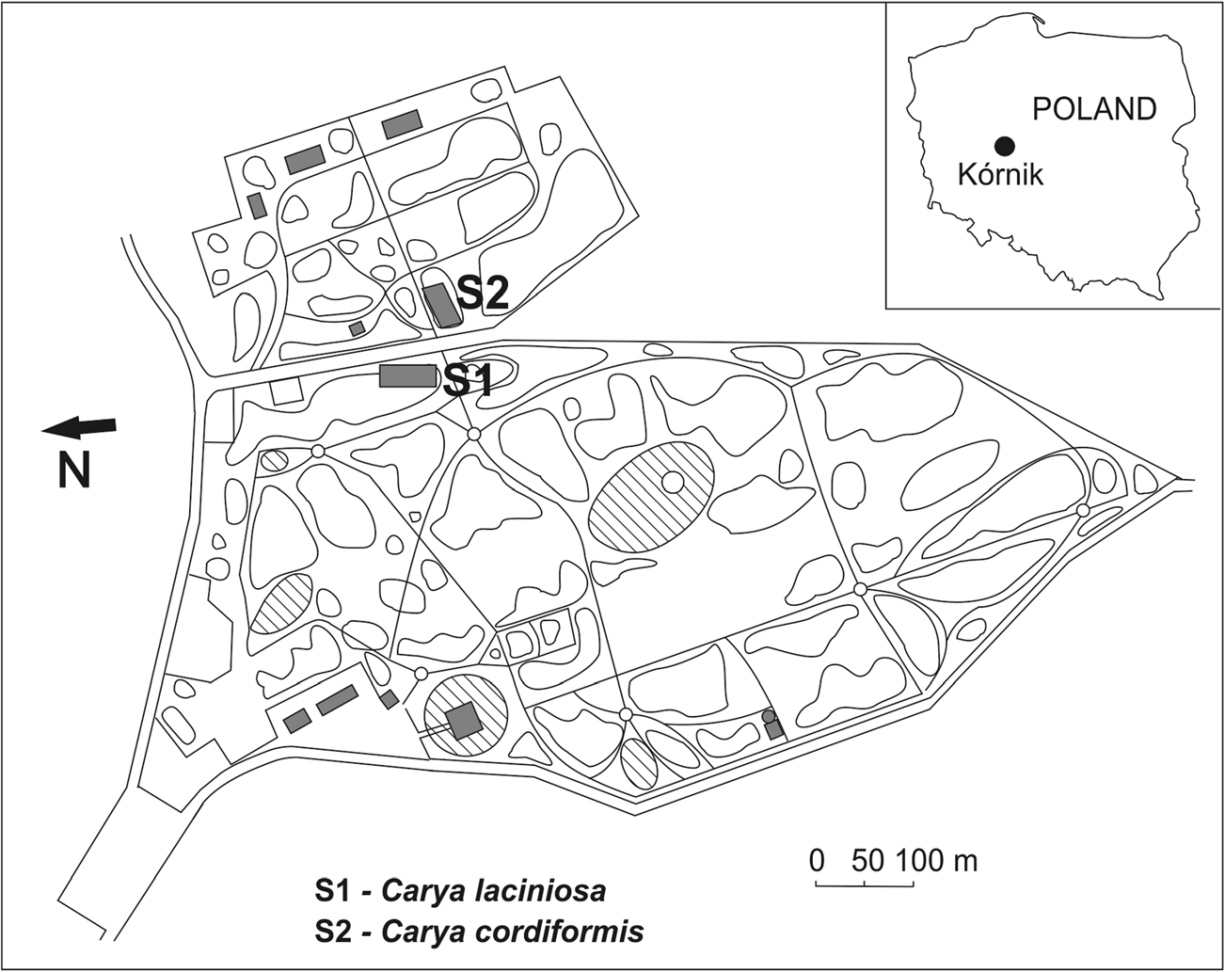
Localization of the study sites on the area of Kórnik Arboretum, Poland

The study was carried out at Kórnik Arboretum, which is located in Western Poland (17° 06′ E, 52° 15′ N), at an altitude of 75 m asl. The study site was selected, from among a few groups of hickories grown in the collections there, on the basis of the occurrence of naturally regenerated seedlings under the canopy of mature hickory trees. The first site of area 250 m^2^ (S1) was situated in the old part of the arboretum, around Kórnik Castle, and the second site of area 300 m^2^ (S2) was situated in the so-called New Arboretum, which is separated from the latter by Parkowa Street (Fig. 1). The hickories on both sites are around 150 years old and belong to the oldest representatives of these trees in Poland, and probably Europe; they originate from the collections founded in the years 1845 (*C. cordiformis*) and 1873 (*C. laciniosa*) (Białobok 1960; Chylarecki 1961). Site S1, with *C. laciniosa*, was represented by trees that have reached heights of 21 to 29 m, and trunk diameters of 35 to 51 cm. Site S2, with *C*.*cordiformis*, was represented by trees reaching 31 m in height and 57 cm in diameter. The ground layer at both sites was dominated by a dense cover of ivy (*Hedera helix*). Some other characters of the study sites, including climatic data from a nearby measurement station, and the composition of overstory and understory tree species grown in the direct vicinity of the tested hickories, are provided in Table 1. The soil pH was determined using soil suspended in water, and 0.5 M potassium chloride. The volumetric soil moisture and soil temperature at each site were monitored every 6 h, from August through October 2016, using HOBO Micro Station H21-002 data loggers equipped with Soil Moisture Smart Sensors (S-SMD-005) and Temperature Smart Sensors S-TMB-002 (Onset) (Table 1).

**Table 1:** Characteristics of the study sites from Kórnik Arboretum, Poland

	Site S1 (*Carya laciniosa*)		Site S2 (*Carya cordiformis*)
Soil type	Muck soil on sandy loam		Muck soil on sands
Thickness of organic layer (cm)	6.5		7.5
${\mathrm{pH}}_{{\mathrm{H}}_20}$	5.83		6.09
pH _KCl_	4.74		5.92
Soil moisture (%)^a^	34.8		28.9
Soil temperature (°C)^a^	13.6		13.3
*T*_ave_ (°C)^b^		10.2	
*T*_min_ (°C)^c^		− 6.6	
*T*_max_ (°C)^d^		23.7	
Precipitation (mm)		594	
Accompanying trees and shrubs	*Pterocarya fraxinifolia* (O), (AM) *Aesculus hippocastanum* (O), (AM) *Thuja plicata* (O), (AM) *Taxus baccata* (O&U), (AM) *Deutzia scabra* (U), (AM) *Acer campestre* (U), (AM) *A. platanoides* (U), (AM) *Carpinus betulus* (U), (EM) *Fagus sylvatica* (U), (EM)		*Picea abies* (O), (EM) *Tsuga canadensis* (O), (EM) *Abies* sp. (O), (EM) *Thuja plicata* (O), (AM) *Taxus baccata* (O&U), (AM) *Acer campestre* (U), (AM) *A. platanoides* (U), (AM) *Tilia cordata* (U), (EM) *Populus alba* (U), (AM&EM) *Carpinus betulus* (U), (EM)

*O* overstory, *U* understory, *AM* arbuscular mycorrhiza, *EM* ectomycorrhiza

^a^Mean values calculated from the period August–October 2016

^b^Mean annual temperature (2007–2016)

^c^Mean temperature of the coldest month (2007–2016)

^d^Mean temperature of the warmest month (2007–2016)

### Root sampling and morphotyping of mycorrhizae

In autumn 2015, to tentatively estimate the mycorrhizal colonization of *C. laciniosa* and *C. cordiformis*, soil samples were taken from beneath the canopy projection of sites S1 and S2 with a soil corer (5-cm diameter) to a depth of 20 cm. The mature hickories appeared, in this arboretum habitat, to be characterized by rather patchy, fine root distributions, which made sampling of active mycorrhizal root tips with the soil corer rather difficult. Additionally, due to the admixture of other trees, we could not be sure that we were only sampling hickory roots. Therefore, in the next year (September 2016), we dug out intact root systems of all *Carya* seedlings (10 seedlings of C. *laciniosa* and 11 seedlings of *C. cordiformis*), naturally regenerated under the canopy of mature *Carya* trees. For the final analysis, six seedlings of each species with the best-developed root systems were selected. We based our approach on the general assumption that mycorrhizal colonization mainly occurs via a common mycorrhizal network resident in the forest soil, resulting in a similar set of EM fungi present on seedlings and surrounding trees (Aučina et al. 2011; Cline et al. 2005; Jonsson et al. 1999). *Carya* seedlings, growing not closer to each other than 5 m, (1–3 years old; 30 to 110 cm high) were cautiously sampled from both sites, using a shovel to take intact soil blocks (20 × 20 × 25 cm depth; including the organic and mineral horizons) and avoiding damage to the root systems. In the laboratory, the roots of each seedling were carefully washed under running tap water. Clean fragments of *Carya* seedling roots were submerged in water on a Petri dish, and examined with a Zeiss Stemi 2000-C stereomicroscope (Carl Zeiss, Germany; ×10–60 magnification) for EM assessment. Depending on the observed turgid state, the mycorrhizae were categorized as either alive or dead. Live ectomycorrhizae were further classified into different, distinguishable groups, using morphotyping (Agerer 1987–2008). Two to five individual EM morphotypes were stored at − 20 °C for molecular analysis. The rest of the root systems were cleared and stained, as described below, to determine the degree of EM and AM fungal colonization.

### Assessment of fungal colonization in roots

Root samples were cut into 1-cm segments, and 0.25-g subsamples were prepared. Individual root subsamples were cleared with 10% potassium hydroxide, first at room temperature (24 h), and later at 96 °C (2 × 40 min). In the next stage, roots were bleached with 10% hydrogen peroxide, and stained with Trypan blue in lactoglycerol (modified from Kormanik and McGraw 1982). Root pieces, mounted on microscope slides in lactoglycerol, were examined at × 100–400 magnification (Zeiss Axio Imager.A1 microscope). One hundred root intersections were examined, and intersections containing arbuscules, vesicles, or internal hyphae were scored as arbuscular mycorrhizae, while root segments covered by a fungal mantle were scored as ectomycorrhizae. Additionally, during the microscopic observations, the percentage colonization by fungal endophytes (FE) was also assessed. From the observed FE, dark septate endophytes were distinguished based on their morphology, i.e., dark hyphal color, thicker lateral wall, frequent septa (Lingfei et al. 2005).

Fungal colonization in the roots is presented as a percentage of root length colonized (%RLC).

### Molecular identification of ectomycorrhizal root tips

The identification of previously selected and preserved morphotypes was based on molecular analysis of two to three mycorrhizal tips of each unique morphotype. Total DNA was extracted using the GeneMATRIX Plant and Fungi DNA Purification Kit (EURx, Poland), following the manufacturer’s protocols. The fungal internal transcribed spacer (ITS) region was amplified with ITS1F/ITS4 primers, using a Type-it Microsatellite PCR Kit (Qiagen, Germany). The PCR products were sequenced at the Laboratory of Molecular Biology in the Adam Mickiewicz University in Poznan, using a CEQ 20000XL automatic sequencer with an ITS4 primer. The obtained sequences were edited using BioEdit 7.2, and compared with reference fungal ITS sequences from the UNITE and GenBank databases (www.ncbi.nlm.nih.gov), using BLAST (Altschul et al. 1990). Species-level identification of the mycorrhizae was defined as sharing > 97% of the ITS region. The best representatives of each unique ITS sequence were deposited in the NCBI GenBank with the accession numbers MG835414-MG835446.

### Statistical analysis

Ectomycorrhizal fungal diversity on tested *Carya* seedlings was evaluated by determining species richness, mean species richness per seedling, relative abundance, and frequency. The relative abundance of fungal taxa was calculated for each seedling separately, by dividing the number of root tips colonized by each identified taxon by the total number of live ectomycorrhizae, and then averaging that for all seedlings from a given *Carya* species. Frequency was calculated as the percentage of seedlings on which each unique EM fungal taxon was found. To evaluate the sufficiency of the sampling effort, the Chao-2 richness estimator was calculated with the EstimateS program v. 9.0 (Colwell 2013), using 100 randomized runs, without sample replacement, with an individual seedling as one sample unit. Differences in the percentage of root length colonization and mean species richness, between both *Carya* species, were tested by one-way analysis of variance (ANOVA). The statistical differences in EM fungal assemblages on different hosts (*C. laciniosa* and *C. cordiformis*) were tested using a one-way analysis of similarities (ANOSIM) and nonmetric multidimensional scaling ordination (NMDS) based on Bray–Curtis similarity coefficient matrix. ANOSIM and NMDS analyses were carried out with PAST 1.89 software (Hammer et al. 2001).

## Results

**Fig. 2:**
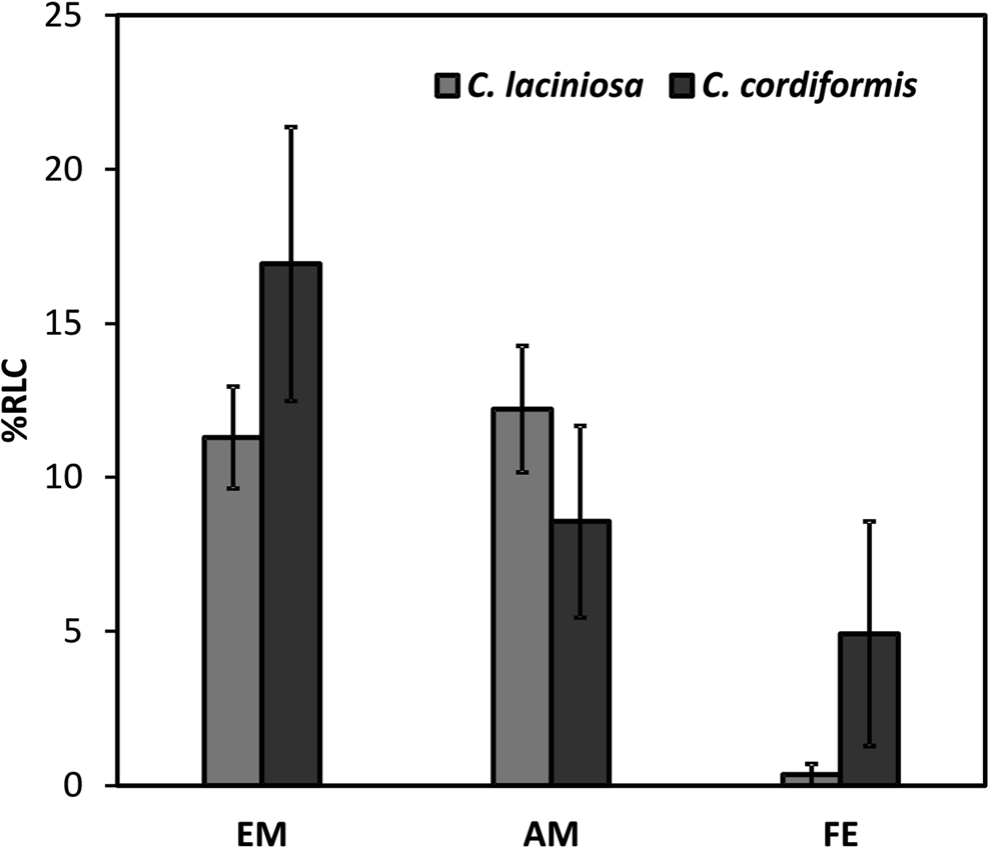
Root length colonization (%) of *Carya laciniosa* and *C. cordiformis* naturally regenerated seedlings, from Kórnik Arboretum, by EM and AM fungi, and fungal endophytes (FE) (mean ± SE)

Dual mycorrhizal colonization by EM and AM fungi was observed on the roots of both *Carya* species. Ectomycorrhizal colonization on the roots of *C. cordiformis* and *C. laciniosa* was found to be 16.9% RLC and 11.3% RLC respectively. The AM colonization of *C. cordiformis* and *C. laciniosa* was 12.2% RLC and 8.6% RLC respectively (Fig. 2). Among the fungal features of arbuscular mycorrhizae, typical structures (vesicles, arbuscules, hyphal coils, and intercellular nonseptate hyphae) were observed in the roots of both *Carya* species (Fig. S1). The hickory roots were also colonized, to different degrees, by FE (Figs 2, S1), with the dark septate fungi prevailing (over 99% of all FE). All observed differences in colonization by EM and AM fungi, and FE between both hickory species were not statistically significant (Fig. 2).

**Fig. 3:**
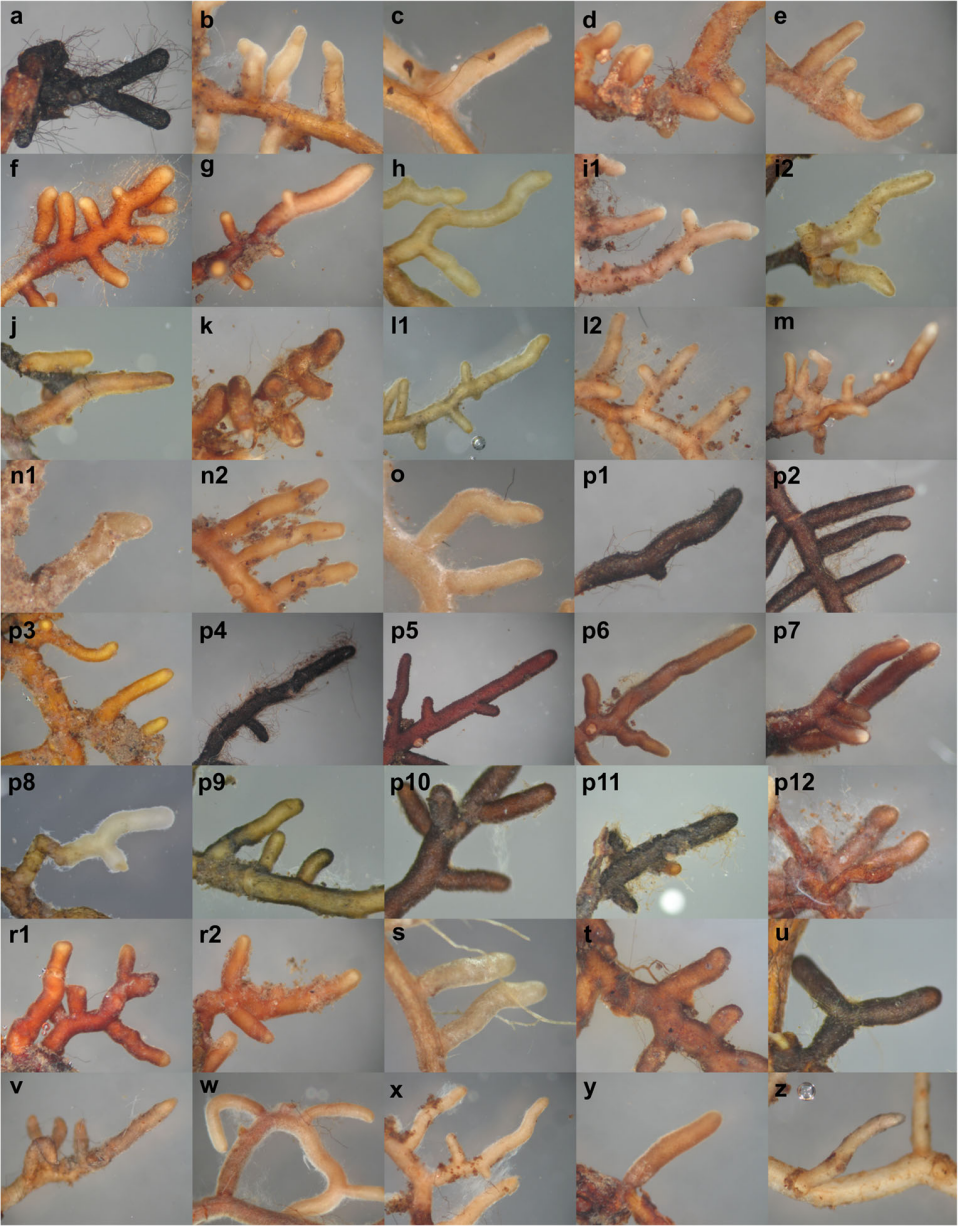
Plan view of mycorrhizae observed on *C. laciniosa* and *C. cordiformis* naturally regenerated seedlings: *Cenococcum geophilum* s.l. (**a**); *Cortinarius subexitiosus* (**b**); *Hebeloma leucosarx* (**c**); *Helvella* sp. (**d**); *Helvellosebacina helvelloides* (**e**); *Humaria hemisphaerica* (**f**); *Hydnobolites* sp. (**g**); *Hydnotrya tulasnei* (**h**); *Inocybe asterospora* (**i1**); *Inocybe pusio* (**i2**); *Laccaria laccata* (**j**); *Melanogaster variegatus* (**k**); *Otidea alutacea* (**l1**); *Otidea bufonia* (**l2**); *Peziza succosa* (**m**); *Russula parazurea* (**n1**); *Russula recondita* (**n2**); *Scleroderma areolatum* (**o**); *Tomentella badia* (**p1**); *Tomentella cinereoumbrina* (**p2**); *Tomentella galzinii* (**p3**); *Tomentella* sp.1 (**p4**); *Tomentella *sp.2 (**p5**); *Tomentella *sp.3 (**p6**); *Tomentella *sp.4 (**p7**); *Tomentella* sp.5 (**p8**); *Tomentella *sp.6 (p**9**); *Tomentella *sp.7 (**p10**); *Tomentella *sp.8 (**p11**); *Tomentella *sp.9 (**p12**); *Tuber *sp.(**r1**); *Tuber rufum* (**r2**); *Xerocomellus cisalpinus* (**s**); Unidentified fungus 1 (**t**); Unidentified fungus 2 (**u**); Unidentified fungus 3 (**v**); Unidentified fungus 4 (**w**); Unidentified fungus 5 (**x**); Unidentified fungus 6 (**y**), Unidentified fungus 7 (**z**)

**Fig. 4:**
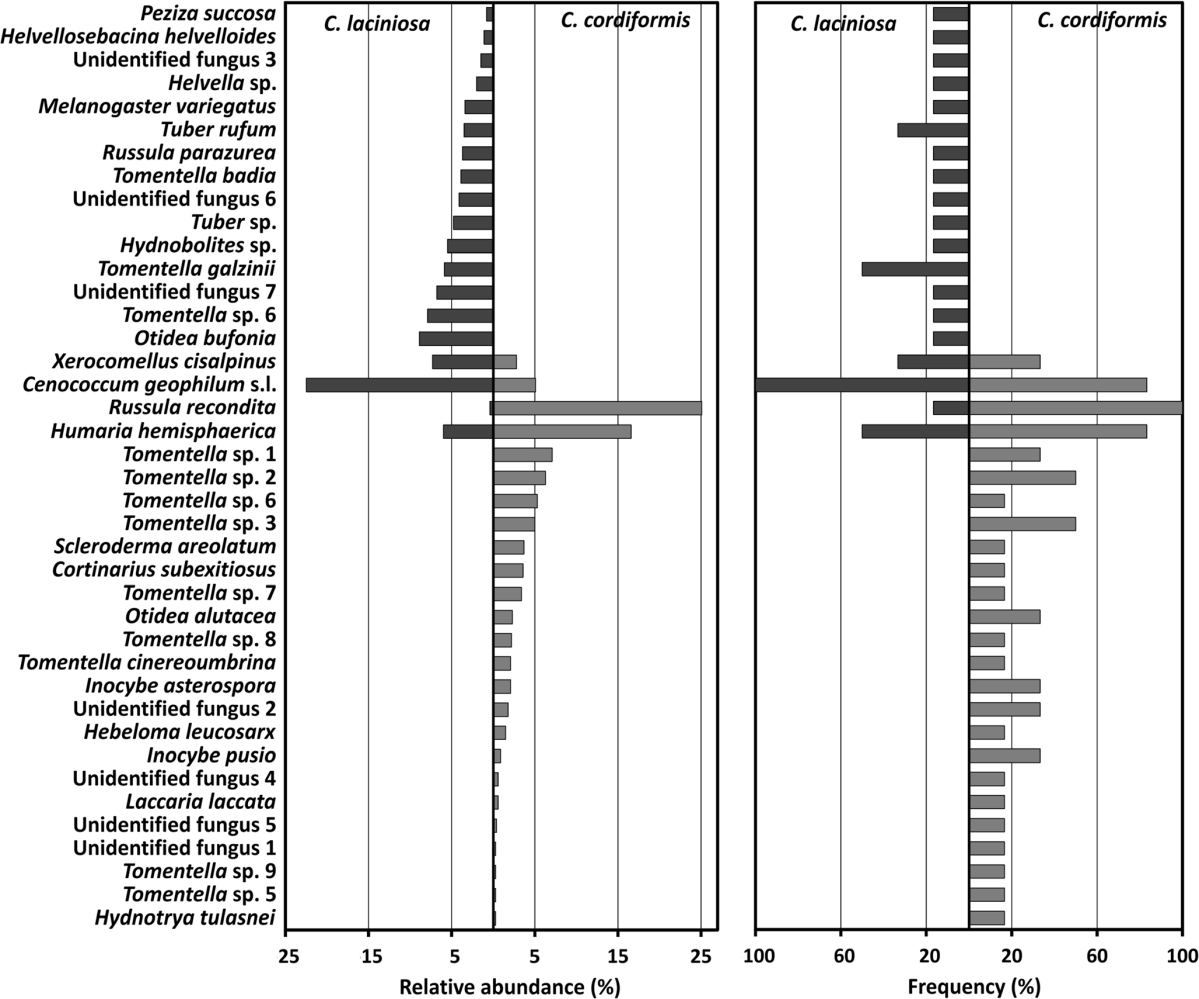
Relative abundance (**a**) and frequency (**b**) of EM fungal taxa associated with *Carya laciniosa* and *C. cordiformis* naturally regenerated seedlings from Kórnik Arboretum, Poland

The morphological assessment conducted on 4293 EM root tips revealed 2927 living ectomycorrhizae. From these root tips, 148 morphotypes were tentatively separated, of which, after regrouping and combining on the basis of the results of the molecular analysis, finally 40 fungal taxa were detected (Table 2, Figs 3, and 4). The richness estimator, Chao-2, indicated that at least 63.7 EM fungal taxa were expected to colonize the seedlings of both *Carya* species. The observed fungal taxa richness was 25 for *C. laciniosa* and 19 for *C. cordiformis*. The calculation of EM fungal taxa richness, based on the Chao-2 estimator, revealed that estimated richness amounted to 32.2 for *C. laciniosa* and 35.3 for *C. cordiformis.* The mean taxa richness per seedling was significantly higher (ANOVA, *P* = 0.031) for *C. laciniosa* (8.0 ± 1.2) than for *C. cordiformis* (4.8 ± 1.5).

**Table 2:** Ectomycorrhizal fungal taxa detected on the roots of naturally regenerated seedlings of *Carya laciniosa* and *C. cordiformis* from Kórnik Arboretum, Poland. Taxa are listed alphabetically

Fungal taxon	Accession No	Closest match	Identity (%)	*E* value	Geographic distribution^†^
*Cenococcum geophilum* s.l.	MG835414	*Cenococcum geophilum* (AM161511)	99.72	0.00	C
*Cortinarius subexitiosus*	MG835415	*Cortinarius subexitiosus* (KP165574)	99.24	0.00	EU, NA
*Hebeloma leucosarx*	MG835416	*Hebeloma leucosarx* (UDB017706)	98.33	0.00	EU, NA
*Helvella* sp.	MG835417	*Helvella* (UDB024235)	99.56	0.00	EU‡
*Helvellosebacina helvelloides*	MG835418	*Helvellosebacina helvelloides* (KF000435)	99.20	0.00	EU, NA
*Humaria hemisphaerica*	MG835419	*Humaria hemisphaerica* (UDB023667)	97.17	0.00	EU, NA
*Hydnobolites* sp.	MG835420	*Hydnobolites* (EU816665)	98.77	0.00	EU‡
*Hydnotrya tulasnei*	MG835421	*Hydnotrya tulasnei* (UDB000095)	99.33	0.00	EU
*Inocybe asterospora*	MG835422	*Inocybe asterospora* (UDB000098)	100.00	0.00	EU, NA, AU
*Inocybe pusio*	MG835423	*Inocybe pusio* (UDB031390)	99.82	0.00	EU, NA
*Laccaria laccata*	MG835424	*Laccaria laccata* (UDB000104)	100.00	0.00	EU, NA
*Melanogaster variegatus*	MG835425	*Melanogaster variegatus* (UDB001487)	97.70	0.00	EU
*Otidea alutacea*	MG835426	*Otidea alutacea* (UDB024230)	99.81	0.00	EU, NA
*Otidea bufonia*	MG835427	*Otidea bufonia* (UDB031339)	99.78	0.00	EU
*Peziza succosa*	MG835428	*Peziza succosa* (UDB015317)	98.36	0.00	EU
*Russula parazurea*	MG835429	*Russula parazurea* (UDB022561)	99.27	0.00	EU
*Russula recondita*	MG835430	*Russula recondita* (KJ530756)	99.49	0.00	EU
*Scleroderma areolatum*	MG835431	*Scleroderma areolatum* (UDB031438)	97.61	0.00	EU, NA, SA
*Tomentella badia*	MG835432	*Tomentella badia* (UDB001656)	97.63	0.00	EU, NA
*Tomentella cinereoumbrina*	MG835433	*Tomentella cinereoumbrina* (UDB016491)	98.28	0.00	EU
*Tomentella galzinii*	MG835434	*Tomentella galzinii* (UDB000263)	98.63	0.00	EU
*Tomentella* sp.1	MG835435	*Tomentella* (UDB018564)	94.79	0.00	EU^‡^
*Tomentella* sp.2	MG835436	*Tomentella stuposa* (UDB002429)	95.51	0.00	EU^‡^
*Tomentella* sp.3	MG835437	*Tomentella* (UDB020340)	98.85	0.00	EU^‡^
*Tomentella* sp.4	MG835438	*Tomentella* (UDB018389)	99.75	0.00	EU^‡^
*Tomentella* sp.5	MG835439	*Tomentella* (UDB022947)	95.00	0.00	SA^‡^
*Tomentella* sp.6	MG835440	*Tomentella coerulea* (UDB016469)	94.52	0.00	EU^‡^
*Tomentella* sp.7	MG835441	*Tomentella lateritia* (UDB000963)	93.02	4e-178	EU^‡^
*Tomentella* sp.8	MG835442	*Tomentella* (UDB025520)	97.39	0.00	AF^‡^
*Tomentella* sp.9	MG835443	*Tomentella* (UDB028240)	99.60	0.00	EU^‡^
*Tuber* sp.	MG835444	*Tuber* (UDB033025)	100.00	0.00	EU^‡^
*Tuber rufum*	MG835445	*Tuber rufum* (UDB027553)	99.17	0.00	EU
*Xerocomellus cisalpinus*	MG835446	*Xerocomellus cisalpinus* (UDB002180)	99.68	0.00	EU
Unidentified fungus 1	n/a*				
Unidentified fungus 2	n/a				
Unidentified fungus 3	n/a				
Unidentified fungus 4	n/a				
Unidentified fungus 5	n/a				
Unidentified fungus 6	n/a				
Unidentified fungus 7	n/a				

^†^Based on locations with available sequence data (PlutoF biodiversity platform): *C* cosmopolitan, *AF* Africa, *AU* Australia, *EU* Eurasia, *NA* North America, *SA* South America

^‡^Based only on location of reference sequence (closest match sequence)

*Not applicable—EM morphotype failed to amplify; identification based on morphology only

From among 40 detected fungal taxa, 22 were assigned to species and 11 were assigned to a genus (Table 2, Fig. 3). The seven morphotypes not identified by molecular analysis were classified as unidentified fungal taxa (Unidentified fungus 1–7, Table 2 and Fig. 3). The best matches for most of identified EM fungi are from Eurasia and none of them match exclusively with sequences from North America (Table 2). *Carya laciniosa* and *C. cordiformis* shared only four species: *Cenococcum geophilum* s.l., *Russula recondita*, *Xerocomellus cisalpinus*, and *Humaria hemisphaerica*. The distribution of these taxa between both hickories was highly differentiated (Fig. 4a, b). *Cenococcum geophilum* s.l. was found to be the most abundant (22.5%) and frequent (100%) EM fungal species on the site of *C. cordiformis*, whereas on the site of *C. laciniosa*, it made up only 5.1% of the ectomycorrhizae, with a frequency of 33%. *Russula recondita* was the most abundant (25.1%) and frequent (100%) EM fungal taxon on the roots of *C. laciniosa.* In contrast, this fungus on *C. cordiformis* colonized only 0.4% of the root samples, with a frequency of 17%. Besides the four EM fungal species in common, the rest of the EM fungal communities were highly uneven between the two analyzed *Carya* species (Fig. 4a, b) and, additionally, were found in low abundances and low frequencies. Fourteen (74%) of the fungal taxa were found only once on *C. cordiformis*, and 14 (56%) of the taxa were found only once on *C. laciniosa* (Fig. 4b). An ANOSIM revealed that differences in mycorrhizal fungal communities among tree *C. laciniosa* and *C. cordiformis* were highly significant (ANOSIM global *R* = 0.76, *P* = 0.001). Consistently, the NMDS ordinations of the mycorrhizal fungal assemblages (final stress = 0.17) showed complete separation based on tree host species (Fig. S2).

## Discussion

Recent interest in maintaining biological diversity has intensified the need to deliver accurate mycological data from a wide range of terrestrial ecosystems (Giachini et al. 2004). The lack of attention to belowground fungal symbionts developed on exotic tree species grown in arboreta provided the major motivation behind this study. It is important to further our general understanding of the extension of the range of exotic trees into new environments. Additionally, we were prompted by the latest findings from orchards of cultivated *C. illinoinensis* in the USA, where high EM fungal diversity was found, with truffle-forming fungi prevailing, in belowground EM fungal communities (Bonito et al. 2011, 2012). The *Carya* species analyzed in our study, from Kórnik Arboretum, belong to the oldest representatives of hickories in Poland, and probably in Europe, and originated from seeds imported by the owners of the arboretum from famous nurseries in Western Europe during the nineteenth century (Chylarecki 1961). In those times, knowledge of mycorrhizal associations was in its infancy, and during the process of *Carya* introduction and selection, the symbiosis was not considered. The great vitality, as well as the strong and healthy development, of the investigated hickory trees suggests, however, that a paucity of mycorrhizal symbionts had not been a problem in the establishment of *C. laciniosa* and *C. cordiformis* in the growth conditions of Kórnik Arboretum. The EM fungal community on both *Carya* species from Kórnik Arboretum yielded 40 different taxa, 25 with *C*. *laciniosa* and 19 with *C. cordiformis.* Thus, our expectation that the EM fungal community would be, to some extent, limited was confirmed, because deciduous trees, when grown in their natural habitats, are generally more species-rich (number of fungal species ranging from 43 on hornbeam to 140 on oaks; Lang et al. 2011; Morris et al. 2008; Suz et al. 2014). The high number of fungal species that were found only once or twice suggests that contacts between roots and many of the rather rare fungal species may be restricted. This assumption also arose from the estimation of EM fungal taxa richness (Chao-2), which indicated that only 77.6% of *C*. *laciniosa* and 53.8% fungal partners of *C. cordiformis* were detected in our survey.

We are not aware of any reports on EM fungal communities associated with *C. laciniosa* and *C. cordiformis*, either from their home range in North America, or from outside of their native range. The only comparison we could perform was an analysis of EM root tips, sampled from a total of 50 individually cultivated *C. illinoinensis* trees from southern Georgia, which revealed 44 distinct EM fungal taxa from 16 genera (Bonito et al. 2011). Differences in overstory tree species compositions, climate, tree age, and sampling strategy make comparisons between the findings of Bonito et al. (2011) and our research rather difficult (Dickie and Reich 2005). However, the composition of the EM fungal community of *C. laciniosa* and *C. cordiformis* from Kórnik Arboretum is rather similar to what is known from *C. illinoinensis*. Ten families and nine genera were found on both American stands and within Kórnik Arboretum (Table S1). As for the species potentially common for those two geographical areas, four *Tomentella*, one *Russula*, and one *Tuber* species (Fig. S3 A-C), as well as *C. geophilum* s.l., were found. Nevertheless, our results clearly indicate that alien-to-Poland *Carya* species were able to form effective mycorrhizae with fungal associates present in the soil environment of Kórnik Arboretum. The assortment of EM fungi colonizing both tested *Carya* species includes many geographically widespread (Table 2) and host-generalist species (Ishida et al. 2007, Lang et al. 2011). The abundance distribution of these species (Fig. 4) represents a typical pattern for EM assemblages in studies of fungal ecology (e.g., Tofts and Orton 1998), which are generally dominated by a few taxa with a “long tail” of relatively rare taxa (Taylor 2002). Unexpectedly, *C. laciniosa* had only four fungal species in common with *C. cordiformis*, namely *C. geophilum* s.l., *R. recondita*, *X. cisalpinus*, and *H. hemisphaerica*. *Cenococcum geophilum* s.l. was found to be the most abundant and frequent EM fungal taxon on the *C. cordiformis* site, a finding common to many other EM fungal surveys in different forest types (Buée et al. 2007; Goodman and Trofymow 1998; Ishida et al. 2007; Lang et al. 2011; Luoma et al. 2006; Pickles et al. 2010). Abundant root colonization by *C. geophilum* s.l. is often considered to be an indicator of drought stress (Pigott 1982) and, in the case of our study, may be the result of competition for water resources, arising from a dense soil cover by *Hedera helix*. *Cenococcum geophilum* was recently phylogenetically analyzed by Obase et al. (2016). Using a combination of ITS ribosomal DNA and the glyceraldehyde-3-phosphate dehydrogenase (GAPDH) gene, six well-supported clades were resolved, two of which are found in Europe. Due to our research being conducted using only the ITS gene analyses, it is not possible to determine which clade (or clades) the *C. geophilum* found in our study belongs to. For this reason, we decided to refer to this taxon as *C. geophilum* sensu lato.

The most abundant and frequent EM fungal taxon on *C. laciniosa* was *R. recondita.* This species was recently separated from *Russula pectinatoides* as a result of taxonomic revision (Melera et al. 2017), suggesting that all of the previous European records of *R. pectinatoides* should be reassigned to *R. recondita*. As *R. recondita* is often found as an EM symbiont of deciduous trees, especially oaks (Lang et al. 2011; Schmit et al. 1999), we presumed that old oaks that thrive in the vicinity of the *Carya* sites in Kórnik Arboretum may be the source of this symbiont for the hickory trees. However, the detailed environmental variables, critical in shaping these fungal communities, remain unknown at this stage of the study. It has been found that plant host species, in a contrasting way, influence EM fungal community composition. Some studies provide evidence that EM fungal community assemblages vary with host species (Cavender-Bares et al. 2009; Morris et al. 2008, 2009), but others show that congeneric tree species can have very similar EM fungal communities (Ishida et al. 2007; Leski et al. 2010; Walker et al. 2005). Differences in tolerance of biotic soil properties, and the specificity of the surrounding tree community, could be partially responsible for differences in patterns of mycorrhizal species community composition in both studied hickory species. Additionally, the differences in species composition found in this study, for the two tested *Carya* species, might also suggest that multi-host EM fungal species still exhibit some host preferences in a given ecological context, or that their competitiveness differs on different host trees (Lang et al. 2011). Obviously, more research on *Carya*’s EM fungal communities remains necessary to determine whether the EM fungal communities of *C. laciniosa* and *C. cordiformis*, grown in Kórnik Arboretum, are attributable to the specific host, or whether there is an important habitat or biogeographical effect.

The hickory trees from this experiment were introduced to Kórnik Arboretum in the nineteenth century as seedlings, produced in a local nursery from seeds imported from Western European companies (Białobok 1961). Hence, at the time of planting, the *C. laciniosa* and *C. cordiformis* seedlings were devoid of their usual fungal symbionts. At the same time, and also in the following decades, extensive intercontinental movement of planting material, with soil containing mycorrhizae, was undertaken to Kórnik Arboretum (Białobok 1960), without paying attention to the potential phytosanitary risk posed by this action. Therefore, we hypothesized that some alien EM fungal species, unintentionally transported with other exotic trees, might be present in the soil environment of Kórnik Arboretum. But, on the tested hickories, we did not find any EM fungal species that could be determined as alien to Europe and native to North America (Table 2). However, at least one species, *Cortinarius subexitiosus*, found as an EM fungus on *C. laciniosa*, merits more attention. Molecular analysis revealed that *C. subexitiosus* is a sister taxon to *C. exitiosus*, deviating from the latter in the ITS regions by 11 base substitutions and indel positions (Niskanen 2014). The fruit body of the holotype of *C. subexitiosus* originated from the USA (Washington), from conifer-dominated (*Tsuga*, *Pseudotsuga*, *Abies*, *Pinus*) forest, intermingled with some *Populus*, *Alnus*, and *Salix*. A detailed description of this fungus was performed by Niskanen (2014), who, for the first time, found fruit bodies of *C. subexitiosus* in 2004 in Finland, in a mixed forest with *Picea abies*, *Betula*, *Populus tremula*, and *Pinus sylvestris* (von Bonsdorff et al. 2015). Recently, fruit bodies of *C*. *subexitiosus* were also found in Poland, in a *Quercus robur* forest (Pietras et al. 2016), and in Spain, in the vicinity of *Corylus avelana* and *Abies alba* trees (Ballarà et al. 2017). Altogether, *C. subexitiosus* seems to be quite widespread, but, because it is macroscopically difficult to distinguish from *C. exitiosus* (Niskanen 2014), it has been mostly overlooked until now and therefore is poorly known. The abovementioned records of *C. subexitiosus* clearly show the ubiquitous occurrence of this fungus under the canopy of different tree species. Our results confirm *C. subexitiosus* as a mycorrhizal partner of *C. laciniosa*.

Seedlings of *C. laciniosa* and *C. cordiformis* from this research also showed a low level of colonization by AM fungi, which, because of their low specificity (Smith and Read 2008), were probably not a limiting factor in the establishment of *Carya* in Kórnik Arboretum. Even low levels of AM fungal colonization are known to be very effective in terms of plant growth response and fast phosphorus uptake (Smith et al. 1998; van der Heijden and Vosatka 1999), and probably contributes significantly to the nutrient demands of the *Carya* trees. The relatively high share of the EM fungi in the mycorrhization of the studied *Carya* seedlings may be related to their occurrence under the canopy of mature *Carya* trees. Mature trees are known to influence and shape the structure of ectomycorrhizae on the surrounding seedlings of the same species (Aučina et al. 2011; Cline et al. 2005; Jonsson et al. 1999). The mycorrhizal community of AM and EM fungi in symbiosis with the same tree was studied in detail on species from the family Salicaceae (Karliński et al. 2010; Neville et al. 2002). The AM species were observed to be the first to establish a symbiotic relation with the plant, gradually being replaced by the EM fungi as the trees aged. Neville et al. (2002) show in their study that in the later tree developmental stages, the two types of mycorrhizae may be present at different depths, as they occupy different niches. EM fungi are more abundant in upper soil layers containing larger air spaces, whereas AM fungi prefer deeper mineral soil layers with small air pores. The ability of the exotic *Carya* species to form mycorrhizae with native AM and EM fungi could be a determining factor in the successful establishment of these tree species in the Kórnik Arboretum habitat.

In conclusion, this research describes, for the first time, the EM fungal community on exotic *Carya* trees grown in Europe, outside their natural range. The conditions in the arboretum were favorable to support colonization of *Carya* with local mycorrhizal fungi. Further research into the EM fungal communities of exotic *Carya* trees, grown on old experimental stands established by German and Austrian foresters in the present boundaries of Poland (Bellon et al. 1977; Białobok and Chylarecki 1965; Schwappach 1901), might further reveal how mycorrhizal fungal communities are structured when the exotic *Carya* trees are surrounded by native forests.

## Electronic supplementary material

Table S1 (PDF 231 kb)

Fig. S1 (PDF 489 kb)

Fig. S2 (PDF 346 kb)

Fig. S3 (PDF 261 kb)
