# Supplementary material for: Mycorrhizal associations of the exotic hickory trees, Carya laciniosa and Carya cordiformis, grown in Kórnik Arboretum in Poland
Source: Mycorrhiza. 2018 Jun 22;28(5):549–60. doi: 10.1007/s00572-018-0846-8 (PMC6182374; doi:10.1007/s00572-018-0846-8)
Supplement: Supplementary file 1 — (PDF 231 kb) [file 572_2018_846_MOESM1_ESM.pdf]

Table S1. The comparison of ectomycorrhizal fungal taxa associated with *Carya laciniosa* and *C. cordiformis* naturally regenerated seedlings from Kórnik Arboretum (Poland) and with *C. illinoinensis* trees from native range in southeast North America (Bonito et al. 2011).

Order	Family	Ectomycorrhizal fungal taxa associated with *Carya* species		
		**Kórnik Arboretum in Poland**		**North America** (Bonito 2011)
		C. laciniosa	***C. cordiformis***	***C. illioinensis***
Agaricales	Cortinariaceae	*Cortinarius subexitiosus*		
	Hydnangiaceae	*Laccaria laccata*		
	Hymenogastraceae	*Hebeloma leucosarx*		*Hebeloma* sp. 1
				Hebeloma sp. 2
				Hymenogaster sp.
				Naucoria sp.
	Inocybaceae	*Inocybe asterospora*		*Inocybe* sp. 1
				Inocybe sp. 2
		Inocybe pusio		*Inocybe* sp. 3
	Tricholomaceae			*Tricholomaceae* 1
				Tricholomaceae 2
Boletales	Boletaceae	*Xerocomellus cisalpinus*		*Boletus* sp.
			Xerocomellus cisalpinus	*Xerocomus* sp.
	Diplocystaceae			*Astreaus* sp.
	Paxillaceae		*Melanogaster ambiguus*	
	Sclerodermaceae	*Scleroderma* sp.		*Scleroderma* sp. 1
				Scleroderma sp. 2
				Scleroderma sp. 3
Cantharellales	Hydnaceae			*Sistotrema* sp.
Eurotiales	Elaphomycetaceae			*Elaphomyces* sp.
	Un Eurotiales			Un *Eurotiales*
Mytilinidiales	Gloniaceae	*Cenococcum geophilum* s.l.	*Cenococcum geophilum* s.l.	*Cenococcum geophilum*
Pezizales	Discinaceae	*Hydnotrya tulasnei*		
	Helvellaceae		*Helvella* sp.	
	Pezizaceae		*Hydnobolites* sp.	*Pachyphleous* sp.
				Peziza sp. 1
				Peziza sp. 2
			Peziza succosa	*Pezizaceae* 2
				Pezizaceae 3
				Pezizaceae 4
	Pyronemataceae	*Humaria hemisphaerica*	*Humaria hemisphaerica*	Un Pyrenomycetaceae
		Otidea alutacea	*Otidea bufonia*	
	Tuberaceae		*Tuber rufum.*	*Tuber lyonii*
			Tuber sp	*Tuber* sp. 47
				Tuber sp. 36
				Tuber sp. 45

Russulales	Russulaceae	*Russula recondita*	*Russula parazurea*	*Russula* sp. 1
				Russula sp. 2
			Russula recondita	*Russula* sp. 3
				Russula sp. 4
				Russula sp. 5
Sebacinales	Sebacinaceae,		*Helvellosebacina helvelloides*	*Sebacina* sp.
Thelephorales	Thelephoraceae	*Tomentella cinereoumbrina*	*Tomentella badia*	*Thelephoraceae* sp. 1
		Tomentella sp. 1	*Tomentella galzinii*	*Thelephoraceae* sp. 2
		Tomentella sp. 2	*Tomentella* sp. 4	*Thelephoraceae* sp. 3
		Tomentella sp. 3		*Thelephoraceae* sp. 4
		Tomentella sp. 5		*Thelephoraceae* sp. 5
		Tomentella sp. 6		*Thelephoraceae* sp. 6
		Tomentella sp. 7		*Thelephoraceae* sp. 7
		Tomentella sp. 8		
		Tomentella sp. 9		
