# Supplementary material for: Mycorrhizal associations of the exotic hickory trees, Carya laciniosa and Carya cordiformis, grown in Kórnik Arboretum in Poland
Source: Mycorrhiza. 2018 Jun 22;28(5):549–60. doi: 10.1007/s00572-018-0846-8 (PMC6182374; doi:10.1007/s00572-018-0846-8)
Supplement: Supplementary file 2 — (PDF 489 kb) [file 572_2018_846_MOESM2_ESM.pdf]

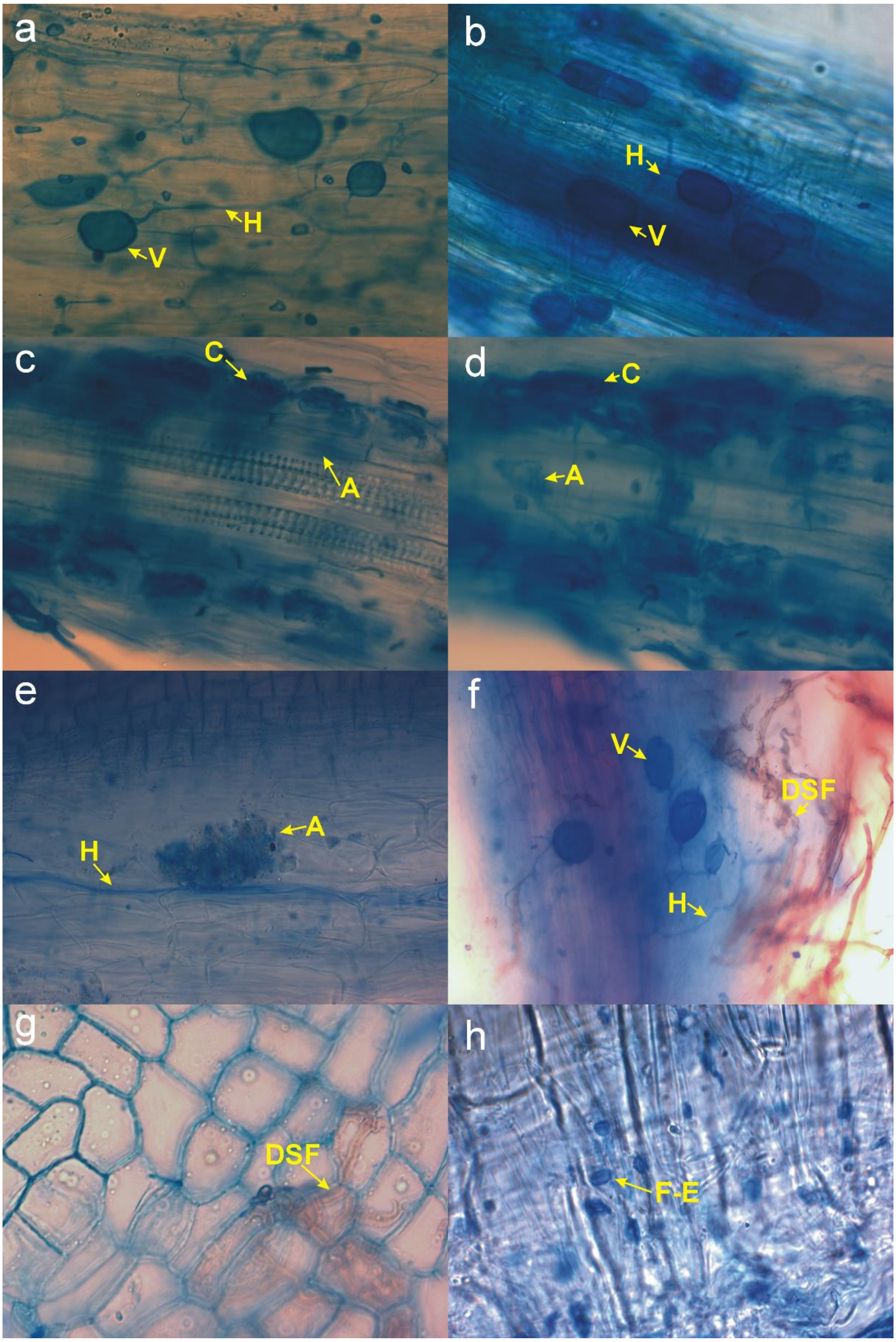

Fig. S1. Structures of arbuscular mycorrhiza (**a-f**) and fungal endophytes (**f-h**) in roots of *Carya laciniosa* and *C. cordiformis* naturally regenerated seedlings from Kórnik Arboretum. **A** – arbuscules, **C** – coils, **H** – hyphae, **V** – vesicles, **DSF** – dark septate fungi (**f** - at the surface of the roots and **g** - inside the roots), **F-E** – other fungal endophytes.
