# Supplementary material for: Mycorrhizal associations of the exotic hickory trees, Carya laciniosa and Carya cordiformis, grown in Kórnik Arboretum in Poland
Source: Mycorrhiza. 2018 Jun 22;28(5):549–60. doi: 10.1007/s00572-018-0846-8 (PMC6182374; doi:10.1007/s00572-018-0846-8)
Supplement: Supplementary file 3 — (PDF 346 kb) [file 572_2018_846_MOESM3_ESM.pdf]

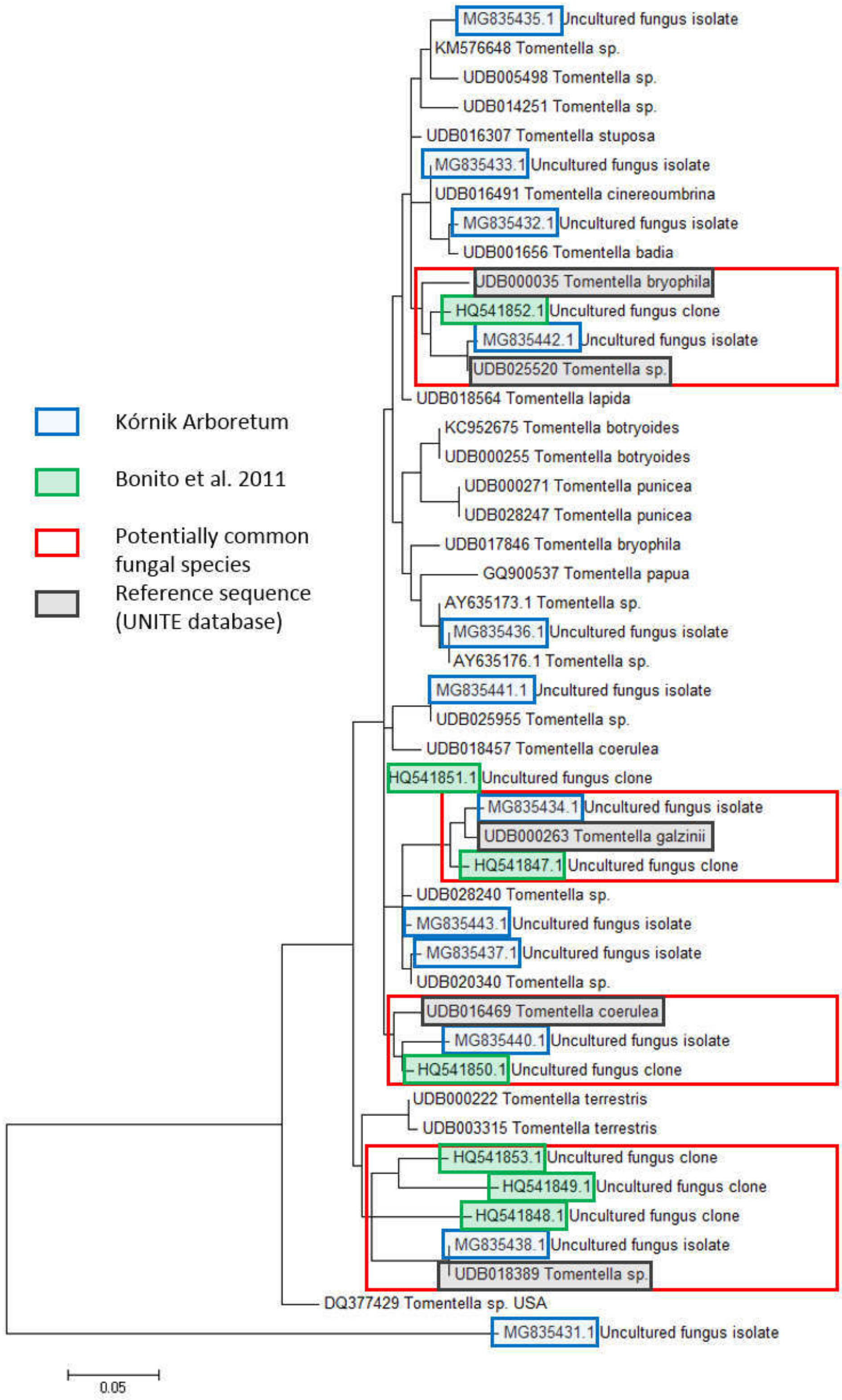

Fig.S3A. Phylogenetic trees of *Tomentella* species identified from *Carya* ectomycorrhizas in Poland (green) and USA (Bonito et al. 2011; blue), on the background of reference sequence (UNITE Database). Species marked red are considered as potentially common for *Carya* in Poland and USA. The phylogram was generated from maximum likelihood method based on ITS-rDNA sequences, used MEGA6 sofware.

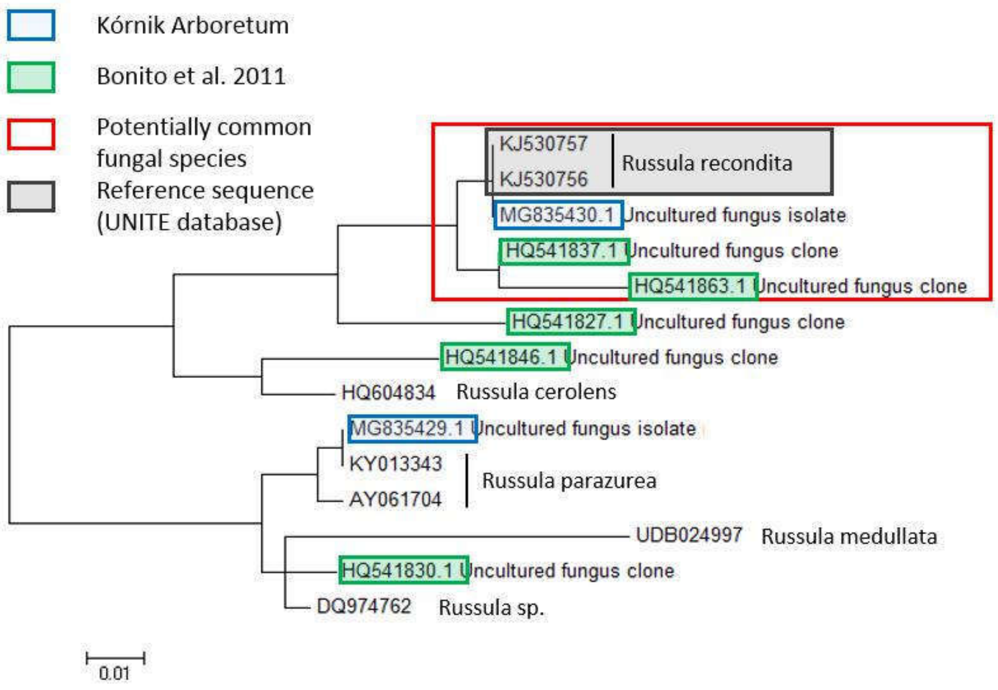

Fig. S3B. Phylogenetic trees of *Russula* species identified from *Carya* ectomycorrhizas in Poland (green) and USA (Bonito et al. 2011; blue), on the background of reference sequence (UNITE Database). Species marked red are considered as potentially common for *Carya* in Poland and USA. The phylogram was generated from maximum likelihood method based on ITS-rDNA sequences, used MEGA6 sofware.

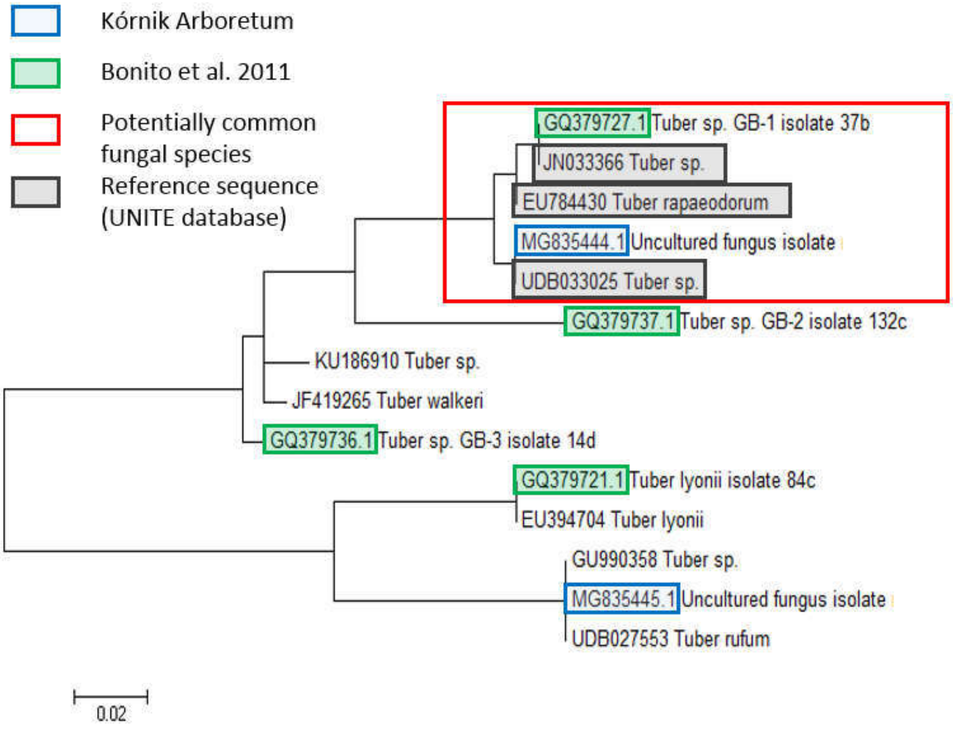

Fig. S3C. Phylogenetic trees of *Tuber* species identified from *Carya* ectomycorrhizas in Poland (green) and USA (Bonito et al. 2011; blue), on the background of reference sequence (UNITE Database). Species marked red are considered as potentially common for *Carya* in Poland and USA. The phylogram was generated from maximum likelihood method based on ITS-rDNA sequences, used MEGA6 sofware.
