# Supplementary material for: Mycorrhizal associations of the exotic hickory trees, Carya laciniosa and Carya cordiformis, grown in Kórnik Arboretum in Poland
Source: Mycorrhiza. 2018 Jun 22;28(5):549–60. doi: 10.1007/s00572-018-0846-8 (PMC6182374; doi:10.1007/s00572-018-0846-8)
Supplement: Supplementary file 4 — (PDF 261 kb) [file 572_2018_846_MOESM4_ESM.pdf]

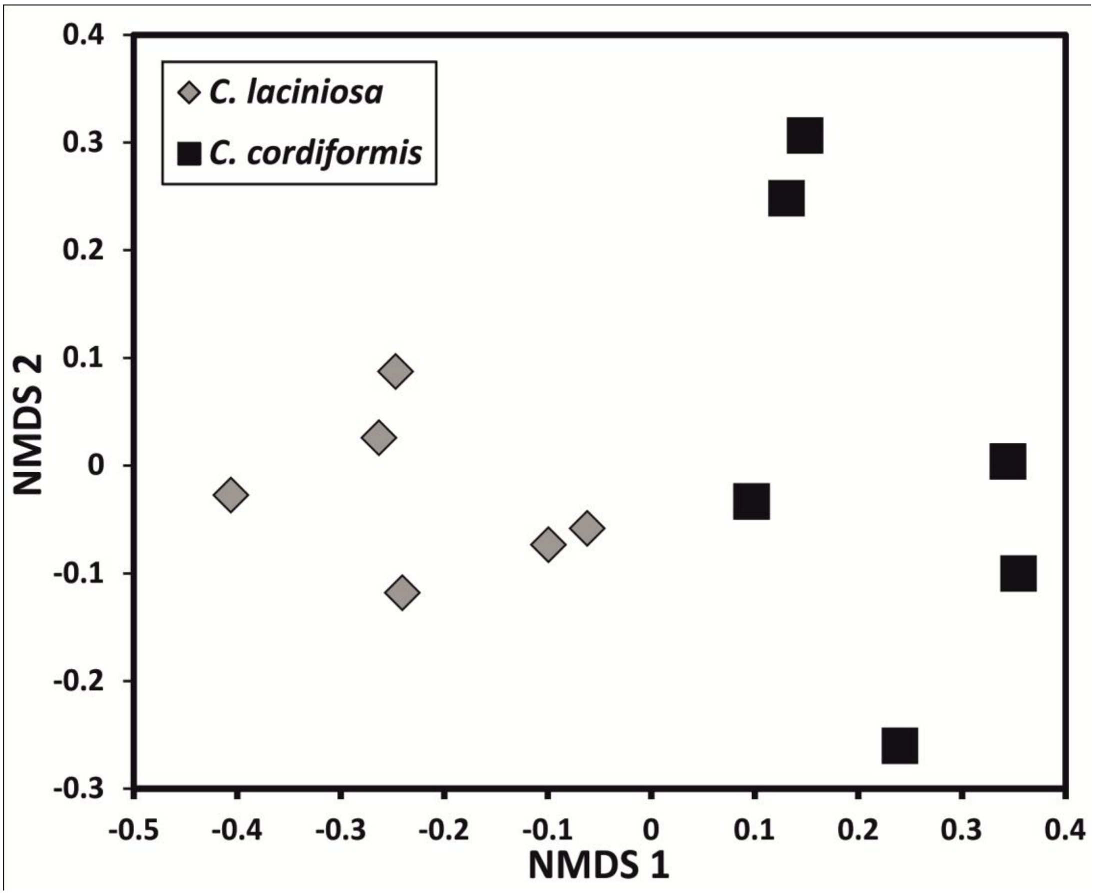

Fig. S2. Two-dimensional nonmetric multidimensional scaling ordination plot of ectomycorrhizal fungal communities, based on Bray–Curtis coefficient. Each point is a representation of ectomycorrhizal fungi composition on naturally regenerated seedlings of *C. laciniosa* and *C. cordiformis* from Kórnik Arboretum, Poland.
